# The development and validation of Coffee Use Disorder and Coffee Addiction Scale (CUDCAS) and its correlation with insomnia and anxiety symptoms

**DOI:** 10.3389/fnut.2025.1674097

**Published:** 2025-11-17

**Authors:** Aseel AlSaleh, Waqar Husain, Hadeel Ghazzawi, Omar A. Alhaj, Achraf Ammar, Khaled Trabelsi, Haitham Jahrami

**Affiliations:** 1 Department of Family and Community Medicine, College of Medicine and Health Sciences, Arabian Gulf University, Manama, Bahrain; 2 Department of Humanities, COMSATS University Islamabad, Islamabad, Pakistan; 3 Nutrition and Food Technology Department, School of Agriculture, The University of Jordan, Amman, Jordan; 4 Department of Nutrition, Faculty of Pharmacy and Medical Sciences, University of Petra, Amman, Jordan; 5 Department of Training and Movement Science, Institute of Sport Science, Johannes Gutenberg-University Mainz, Mainz, Germany; 6 Research Laboratory, Molecular Bases of Human Pathology, LR19ES13, Faculty of Medicine of Sfax, University of Sfax, Sfax, Tunisia; 7 Research Laboratory Education, Motricité, Sport et Santé, EM2S, LR19JS01, High Institute of Sport and Physical Education of Sfax, University of Sfax, Sfax, Tunisia; 8 Department of Movement Sciences and Sports Training, School of Sport Science, The University of Jordan, Amman, Jordan; 9 Government Hospitals, Manama, Bahrain; 10 Department of Psychiatry, College of Medicine and Health Sciences, Arabian Gulf University, Manama, Bahrain

**Keywords:** coffee use disorder, coffee addiction, caffeine consumption, psychometric assessment, scale development, insomnia, anxiety

## Abstract

**Background:**

Coffee is a globally consumed beverage. However, the impairments associated with its excessive use remain under-recognized. There is currently no standardized measurement for coffee use disorder based on DSM-5 application. This study describes the development and psychometric properties of the Coffee Use Disorder and Coffee Addiction Scale (CUDCAS)—a self-report tool developed specifically for this purpose.

**Methods:**

A cross-sectional survey was designed and delivered to 523 participants. Items from CUDCAS (11 items with reference cluster) indicate substance use disorder criteria taken from the DSM-5 and were rated on a three point Likert scale and used descriptive statistics; internal consistency (Cronbach's α and McDonald's ω); exploratory/confirmatory factor analysis (EFA, CFA); item response theory (IRT), and correlations with caffeine consumption, insomnia (AIS) and anxiety symptoms (GAD-7) were also examined.

**Results:**

CUDCAS was found to be a very reliable (α and ω = 0.86) measure of coffee use disorder symptoms. CFA results supported the unidimensional factor structure of the CUDCAS and the overall model fit was good (CFI = 0.92, TLI = 0.90, RMSEA = 0.07, SRMR = 0.04). The IRT analyses further demonstrated an appropriate distribution of item difficulties, measurement of item precision and subsequently, CUDCAS as an overall measurement of coffee use disorder that is responsive to coffee consumption. CUDCAS also demonstrated significant correlations with caffeine consumption (r = 0.54), insomnia (r = 0.37), and anxiety (r = 0.32), respectively, for construct validity.

**Conclusions:**

The findings suggest that the CUDCAS is a reliable and valid tool to assess the symptoms of coffee use disorder, and the current results provide support for its use in research and clinical settings.

## 1 Introduction

Caffeine, a ubiquitous psychoactive substance (1–3), is the most widely consumed stimulant globally (4–8), with its consumption witnessing a significant worldwide increase (9), particularly in regions such as the Middle East and North Africa (10). Despite its pervasive use, caffeine is rarely considered a problematic drug (11–13), even though evidence suggests that for some individuals, caffeine use exhibits characteristics of substance abuse, leading to psychological and physical dependence akin to other addictive substances (14). This often-overlooked aspect of caffeine consumption underscores a critical need for specific and validated tools to identify problematic use and assess its impact on individuals (15).

In the past decade, extensive empirical data has examined the relationship between coffee consumption and psychological outcomes (i.e., anxiety, insomnia). A recent meta-analysis (16) revealed a strong association between caffeine consumption and risk of anxiety—mainly at high doses (≥400 mg)—with standardized mean differences (SMD) of approximately 2.86 (95% CI 2.50–3.22) for high-dose exposure, and 0.61 (95% CI 0.42–0.79) compared to controls at low doses (16). Recent observational evidence in 2025 confirmed this assertion at higher levels of caffeine consumption. Heightened caffeine consumption was associated with higher levels of psychological distress even if this was not directly related to insomnia. This demonstrated more nuanced pathways between consumption behaviors, and emotional wellbeing (17). There have since been further developments in the area of healthy mood dynamics—regular caffeinated beverage drinkers reported significant mood-enhanced ratings on mornings they had coffee compared to mornings they had no caffeine, especially situational mood enhancement effects. Population studies are still confirming high coffee intake is commonly associated with perceived insomnia—up to 54.5% of adults in Jazan, Saudi Arabia, reported adverse sleep impacts associated with coffee use (18).

The recognition of problematic caffeine use as a clinically significant disorder has been a subject of ongoing discussion within the psychiatric community (19, 20). While the 11th Revision of the International Classification of Diseases (ICD-11) acknowledges caffeine withdrawal, caffeine intoxication, caffeine-induced insomnia, and caffeine-induced anxiety disorder as potential diagnoses leading to clinically significant impairment or distress, it does not recognize Caffeine Use Disorder (CUD) as a standalone diagnosis (21). Instead, problematic caffeine use falls under “Other specified disorders due to use of caffeine” or a “harmful pattern of use of caffeine” (21). Historically, the ICD-10 did include substance dependence due to caffeine (22). The Diagnostic and Statistical Manual of Mental Disorders [DSM-5; (23)] provisionally included Caffeine Use Disorder in its Appendix III, titled “Emerging Measures and Models” (23). This classification was intended to stimulate further research into CUD before it could be considered a full clinical diagnosis, largely due to a lack of comprehensive data on its prevalence and clinical implications in the general population. The subsequent DSM-5-TR (85) maintains CUD in Section 3, continuing to emphasize the necessity for further investigation into its validity, reliability, prevalence, and clinical meaningfulness, particularly concerning its impact on functional outcomes (23).

ICD-11 does not use Caffeine Use Disorder as a separate diagnostic category. Nevertheless, ICD-11 recognizes caffeine dependence and caffeine use as a harmful pattern of consumption. These two conditions are clinically valid and share many features of substance use disorder as defined by DSM-5 (23). Specifically, DSM-5 stipulates 11 criteria for substance use disorders, including impaired control, use in a risky way, use leading to social/functional impairment, and physiological aspects such as tolerance and withdrawal (23). ICD-11 describes impaired control, priorities, continuation despite harm, and physiological dependence with harmful consequences (21). Thus, ICD-11 and DSM-5 have similar conceptual frameworks. When the Coffee Use Disorder and Coffee Addiction Scale (CUDCAS) was created, we translated each of the 11 core DSM-5 criteria into items. This provided full coverage of the symptomatic space as well as compatibility with thresholds in ICD-11 for harmful and dependent caffeine use.

The provisional status in the DSM-5 and DSM-5-TR, however, does not negate the clinical relevance of problematic caffeine consumption. Research indicates that caffeine use can play a contributory role in various psychiatric disorders, including anxiety disorder, panic disorder, and major depressive disorder (14, 19, 24–26). Despite this, medical professionals, including doctors and psychiatrists, have historically been reported to rarely inquire about patients' caffeine use (27). This lack of inquiry poses a significant concern, potentially leading to misdiagnosis of caffeine-related disorders and a failure to identify caffeine as a psychoactive substance implicated in a range of psychiatric conditions. For example, studies have shown that problematic caffeine use is associated with higher scores on measures of negative affect, such as depression, anxiety, and stress (14, 19, 24–26). Furthermore, population-based evidence indicates that a notable percentage (8%) of non-clinical adults meet the proposed DSM diagnostic criteria for CUD, with even higher prevalence rates (72–84%) observed among individuals actively seeking treatment for problematic caffeine consumption (28). Fulfilling CUD criteria has been linked to caffeine-related functional impairment, increased psychological distress, and poorer sleep (28).

In addition to its psychological and behavioral impacts, there are negative health effects of coffee addiction that require long-term clinical review. High-frequency, frequent coffee intake may trigger cardiovascular incidents, such as an increase in blood pressure, an increase in heart-rate variability, and the development of arrhythmias, especially among vulnerable groups (29, 30). Another vital issue is gastrointestinal disorders; the coffee addiction has been linked to gastroesophageal reflux disease (GERD), gastritis, and flare-up of peptic ulcer disease, mainly by the way that it stimulates the secretion of gastric acid (31, 32). Bone health impairment is also reported, and the proliferation of coffee consumption will hinder calcium absorption and aggravate urinary elimination of calcium serving to increase the risk of osteoporosis, especially in women beyond menopause (33, 34). Moreover, caffeine dependency may lead to hormonal imbalances, causing disruptions in cortisol patterns and potentially affecting reproductive system hormones, which can impact fertility and menstrual patterns (35, 36).

Given the ubiquity of caffeine use and the potential for over diagnosis if appropriate screening methods are not employed, there is a clear imperative to develop and validate accurate screening and diagnostic tools for CUD. Prior to the studies discussed, existing scales for caffeine-related disorders, such as the Caffeine Craving Scale (37) and the Caffeine Withdrawal Symptom Questionnaire (38), measured only specific aspects of caffeine-related issues. There was no comprehensive measure specifically designed for screening Caffeine Use Disorder for use by medical professionals (1).

In response to this critical gap, two significant self-report instruments have been developed and assessed: the Effects of Caffeine Use Scale (ECUS) (39) and the Caffeine Use Disorder Questionnaire (CUDQ) (15). The ECUS was developed with a rational-empirical approach, constructing a pool of 19 items designed to address the three diagnostic criteria for CUD as prescribed by the DSM-5: persistent desire or unsuccessful efforts to control caffeine use, continued caffeine use despite knowledge of having persistent physical or psychological problems exacerbated by the substance, and withdrawal symptoms (39). An example item for persistent desire/unsuccessful control is: “I have tried to eliminate caffeine from my diet at least once”. For continued use despite knowledge of problems: “On occasion my caffeine intake has interfered with my ability to function effectively”. For withdrawal: “I have consumed caffeine to avoid unpleasant side effects such as headaches, fatigue or lack of motivation”. This scale, initially comprising 19 items (with one later removed due to lexical ambiguity, resulting in 18 items for analysis), demonstrated very high internal reliability (α = 0.94) and adequate concurrent validity through its significant positive correlation with the Depression, Anxiety and Stress Scales (DASS-21). The ECUS was explicitly intended as a short screening tool for medical professionals to detect CUD (39).

Similarly, the CUDQ, developed by Ágoston et al. in 2018 (15), was also based on the proposed CUD criteria from the DSM-5, incorporating nine criteria along with a tenth item assessing suffering caused by caffeine-related symptoms over the last 12 months. This 10-item questionnaire has undergone successful translation, adaptation, and validation in multiple languages, including Turkish (40), Persian (41), and Arabic (42), consistently showing strong psychometric characteristics. The recent validation of the Arabic CUDQ, for instance, conducted in a large community sample (*N* = 1,858) of Arabic-speaking adults, supported a unidimensional factor structure with excellent composite reliability (Cronbach's α = 0.90) and demonstrated good concurrent validity through its positive correlations with nicotine dependence, depression, and anxiety scores (42). The development and validation of scales such as the ECUS and CUDQ are crucial steps in advancing the field. These tools provide a structured and empirically supported means to accurately evaluate problematic *caffeine* consumption, moving beyond subjective estimates of daily intake, which can be inaccurate due to wide variations in caffeine concentration across different beverages and foods. By offering validated instruments, researchers can gather more precise data on the prevalence and severity of CUD across diverse populations and cultural contexts, which is currently lacking. Moreover, these scales empower clinicians to more effectively screen and monitor individuals who may be experiencing difficulties with their *caffeine* use, aiding in the correct recognition of DSM-defined criteria for CUD and informing the development and implementation of targeted treatment opportunities for those who seek assistance to reduce or quit *caffeine* consumption.

However, while the development of the ECUS and CUDQ effectively addresses the need for tools to assess problematic *caffeine* use, a specific gap remains in the literature regarding a dedicated scale to measure “Coffee Use Disorder” and “Coffee Addiction.” The existing scales, while valuable for identifying Caffeine Use Disorder across various sources of caffeine, do not solely focus on coffee. For example, the ECUS instructions explicitly state that “Caffeine is found in coffee, tea, caffeinated soft drinks... energy drinks... cocoa, chocolate and medications” (39). Similarly, the CUDQ measures problematic *caffeine* use generally, even though the validation study mentions “cups of coffee/per day” in its findings; the questionnaire items are universal to caffeine (15). This represents a significant gap because coffee, by its very nature, possesses distinct properties that extend beyond its caffeine content (3, 43, 44). Coffee is not just a source of caffeine; it is a complex chemical mixture containing various biologically active constituents such as minerals, vitamins, lipids, alkaloids, carbohydrates, phenolic, and nitrogenous compounds, which contribute to its unique physiological and psychological effects (2, 45, 46). Furthermore, in many cultures, particularly in Arab countries (47), coffee holds profound cultural, social, and symbolic significance, embodying generosity, nobility, hospitality, and forming a core part of “national mentality” and daily rituals such as weddings, gatherings, and funerals (10). These social and cultural dimensions, alongside the potential synergistic effects of its diverse chemical composition, may lead to unique patterns of problematic use or “addiction” that are specific to coffee and might not be fully captured by a generalized *caffeine* use disorder scale. Therefore, while comprehensive *caffeine* scales are invaluable, there is still a need for a specific scale that accounts for the distinct chemical properties, cultural embeddedness, and social rituals associated with *coffee* consumption, allowing for a nuanced understanding and measurement of “Coffee Use Disorder” and “Coffee Addiction” as separate or more specific constructs. Therefore, the Coffee Use Disorder and Coffee Addiction Scale (CUDCAS) was developed and validated in the current study.

## 2 Methods

### 2.1 Development of the CUDCAS

The development of the CUDCAS followed internationally recognized principles of scale construction rooted in psychometric theory and diagnostic conceptualization (48). The process was guided by a multidisciplinary team comprising nutritionists, psychometricians, psychiatrists, and psychologists to ensure comprehensive expertise in the domains of dietary behavior, scale development, and mental health diagnostics. The first step in the scale's development involved defining the construct. The core constructs in this regard were Coffee Use Disorder and Coffee Addiction. These were clearly delineated based on the DSM-5 criteria for substance use disorders (23). This theoretical grounding ensured content relevance and diagnostic fidelity. The further process involved item generation in which 11 distinct items were formulated to represent each of the 11 DSM-5 symptom domains. Care was taken to tailor these items to coffee-specific behaviors while maintaining theoretical alignment with the broader construct of substance use disorder. The items were written as simple, first-person statements to enhance clarity and ecological validity. To ensure content representativeness, the instructions explicitly guided respondents to report on their coffee consumption over the past 3 months, excluding periods of culturally or contextually abnormal intake such as during religious fasting (e.g., Ramadan), occupational stressors (e.g., exams), or social festivities. This approach helped anchor the responses in habitual patterns rather than situational anomalies. It is a crucial step in minimizing measurement error.

Prior to scale development, we conducted a pilot phase in which semi-structured interviews were held with both experts in substance use research (*n* = 5) and self-identified heavy coffee consumers (*n* = 10). These experts, all of whom held doctoral degrees (PhDs), represented diverse disciplines including psychiatry, clinical psychology, nutrition, and psychometrics. Each brought more than two decades of professional expertise, particularly in the domain of psychometric research and applied practice. They had extensive experience on the design, validation, and implementation of psychological scales and diagnostic tools across varied clinical and research settings. Heavey coffee consumption was considered to consume more than 1,000 milligram or 1 gram coffee per day, as established in an earlier study (49). These interviews provided insight into coffee-specific symptomatology, such as ritualized craving at particular times of the day and the functional consequences of sleep disturbance. Feedback from these interviews was incorporated into the phrasing and contextualization of items, ensuring that the final 11-item pool captured both DSM-5 substance use disorder criteria and coffee-specific experiential nuances.

CUDCAS assesses participants' coffee consumption patterns over the past 3 months. This self-report instrument is designed to measure symptoms indicative of Coffee Use Disorder and Coffee Addiction. The instructions for the CUDCAS explicitly guide participants to consider their coffee consumption habits, while excluding periods influenced by social (e.g., festive holiday like Eid), occupational (e.g., exams or sport competitions), or religious (e.g., Ramadan fasting) practices. While the scale primarily focuses on drinking coffee, its scope also extends to include the use of lozenges and dissolvable coffee pouches.

The CUDCAS comprises 11 items, each corresponding to a specific symptom derived from the DSM-5 criteria for substance use disorders. Participants respond to each statement by indicating how often it applies to them using a three-point Likert-style scale: “Never,” “Sometimes,” or “Always”.

The items and their corresponding DSM-5 symptom domains are as follows:

- “I drink more coffee or consume it in stronger versions” (Larger Amounts).
- “I have tried to cut down or stop drinking coffee, but I can't” (Unsuccessful Attempts to Quit).
- “I spend a lot of time getting coffee, drinking it, or recovering from its effects/side effects” (Time Spent).
- “I feel strong cravings and urges to drink coffee” (Cravings).
- “My coffee drinking interferes with my responsibilities at work, home, or school e.g. late arrival to work due to stopping at café to drink or pick coffee” (Neglecting Responsibilities).
- “I continue to drink coffee even when it causes problems in my relationships e.g. arguments with others who express concern about your excessive coffee consumption” (Relationship Issues).
- “I have given up important activities because of my coffee consumption e.g. adequate sleep or healthy diet” (Giving Up Activities).
- “I drink coffee even when it puts me in risky situations e.g., drinking too much caffeine” (Dangerous Use).
- “I continue drinking coffee despite knowing it worsens physical or psychological issues” (Continued Use Despite Problems).
- “I need to drink more coffee to feel its effects” (Tolerance).
- “I experience withdrawal physical or psychological symptoms that are relieved by drinking coffee” (Withdrawal).

Scoring of the CUDCAS is conducted by assigning numerical values to each response: “Never” is scored as 0, “Sometimes” as 0.5, and “Always” as 1. A total score is calculated by summing the scores of all 11 items.

The suggested interpretation of the total score categorizes the severity of Coffee Use Disorder:

- Mild Coffee Use Disorder: A total score representing < 3 endorsed symptoms.
- Moderate Coffee Use Disorder: A total score reflecting 4 to 6 endorsed symptoms.
- Severe Coffee Use Disorder: A total score indicating 7 or more endorsed symptoms.

### 2.2 Instruments

#### 2.2.1 Coffee intake

Coffee intake was quantified using a previously tested semi-quantitative food frequency questionnaire (FFQ) tailored to assess the consumption of 38 caffeine-containing items commonly consumed. The self-administered questionnaire captured detailed data on the type, serving size, and frequency of consumption for items including regular and decaffeinated coffee, concentrated coffee (e.g., Arabic coffee, Turkish coffee, and espresso), black and green tea, cocoa, energy drinks, soft drinks, chocolates, gums, and over-the-counter caffeine-containing analgesics. Daily coffee intake was calculated by integrating reported consumption patterns with established caffeine content values sourced from the United States Department of Agriculture Nutrient Database (release 28), product labels, and authoritative online sources. This approach enabled a comprehensive estimation of total caffeine intake from coffee (49).

#### 2.2.2 Generalized Anxiety Disorder Scale-7 (GAD-7)

The GAD-7 (50) is a widely used self-report instrument. It is designed to assess the severity of generalized anxiety symptoms in clinical and research settings. It comprises seven items that align with DSM-IV criteria for generalized anxiety disorder. Items are scored on a 4-point Likert scale ranging from 0 (“not at all”) to 3 (“nearly every day”), yielding a total score from 0 to 21. The scale demonstrates excellent internal consistency with Cronbach's alpha typically reported between 0.89 and 0.92 across general and clinical populations (50, 51). Test–retest reliability over a one-week period was also high (intraclass correlation = 0.83) (50). In terms of construct validity, the GAD-7 shows strong correlations with other measures of anxiety and depression (51). The scale's unidimensional factor structure has been confirmed across diverse cultural samples, further establishing its factorial validity (52).

#### 2.2.3 Athens Insomnia Scale (AIS)

The AIS (53) is a psychometrically validated self-report instrument designed to assess the severity of insomnia symptoms in accordance with the ICD-10 diagnostic criteria for insomnia. It consists of eight items, with the first five assessing nocturnal sleep difficulties (such as sleep induction, awakenings during the night, and early morning awakening), and the remaining three evaluating daytime dysfunction (including wellbeing, functioning, and sleepiness). Each item is rated on a 0–3 Likert scale, yielding a total score range from 0 to 24. A cut-off score of ≥6 is commonly used to indicate the presence of clinically significant insomnia (53). The AIS has demonstrated excellent internal consistency, with Cronbach's alpha values typically exceeding 0.84 (54). Construct validity is supported by high correlations with other established sleep assessment tools, such as the Pittsburgh Sleep Quality Index and Insomnia Severity Index, confirming its convergent validity (55). Factorial validity has been supported across multiple language versions, consistently confirming a two-factor structure representing nocturnal symptoms and daytime consequences (56, 57). Its diagnostic utility has been validated in both clinical and non-clinical populations, across diverse cultural contexts (54).

### 2.3 Ethical considerations

This research was conducted according to recognized ethical standards and guidelines, including the Declaration of Helsinki (1964) and its subsequent amendments to protect the rights, dignity, and welfare of all participants. The Research Ethics Committee of the Psychiatric Hospital/Government Hospitals, Bahrain code: REC/PSYGH/2025-9 and date: 5 January 2025. Participation was voluntary, and informed consent was sought from each participant prior to enrolment. All participants had an explanation of the purpose of the research, were assured of the confidentiality of their responses, and were told that their participation is voluntary and they could opt out at any time without penalty. Anonymity was paramount; no identifiable personal information was ever collected or allowed to be linked to participants. No deception was involved in this study; participants also did not incur any foreseeable risks or harm by participating. Data was securely stored and utilized only for research purposes; during all aspects of the study, participants' privacy and confidentiality were ensured.

### 2.4 Data analysis

The obtained data was systematically recorded and analyzed using the R statistical computing environment (Version 4.5.1, released on June 13, 2025, and nicknamed “Great Square Root”). Upon completion of data collection, a rigorous data cleaning process was employed, which included looking for missing values, careless or inattentive responses, outliers, and violations of important statistical assumptions such as linearity, homoscedasticity, multicollinearity, skewness, and kurtosis.

The CUDCAS was evaluated for its psychometric properties using both Exploratory Factor Analysis (EFA) and Confirmatory Factor Analysis (CFA). The EFA was performed through promax rotation with maximum likelihood. The output of the EFA included analyzing the factor loadings, extraction values, Bartlett's Test of Sphericity (BTS), Kaiser-Meyer-Olkin (KMO) measure of sampling adequacy, as well as model fit indices such as Comparative Fit Index (CFI); Tucker–Lewis Index (TLI), Root mean square error of approximation (RMSEA), and Standardized root mean square residual (SRMR). For CFA, we employed maximum likelihood estimation without rotation and a comprehensive model fit evaluation consisted of testing several fit indices, including CFI, TLI, Akaike Information Criterion (AIC), and Bayesian Information Criterion (BIC). Internal consistency was measured through Cronbach's alpha and McDonald's omega.

We also applied Rating Scale Model with Delta-Tau parameterization of the Partial Credit Model, estimated via Marginal Maximum Likelihood Estimation (MMLE), which is based on Item Response Theory (IRT) (58). This analysis further assessed the psychometric properties of the CUDCAS through item functioning, response category ordering, and item fit to the underlying latent trait of coffee use disorder. It allowed for a more precise determination of item difficulty, person reliability, and measurement invariance of the CUDCAS.

Gender invariance of the CUDCAS scale was examined using multi-group confirmatory factor analysis (MGCFA) to assess whether the scale functions equivalently across male and female participants. Three levels of measurement invariance were tested sequentially following established guidelines. First, configural invariance was examined by fitting the same factor structure to both gender groups simultaneously while allowing all parameters to vary freely between groups. Second, metric invariance (weak invariance) was tested by constraining factor loadings to be equal across groups while allowing intercepts and residual variances to vary. Third, scalar invariance (strong invariance) was assessed by additionally constraining item intercepts to equality across groups. Model fit was evaluated using multiple indices, including the chi-square test, CFI, TLI, RMSEA, and SRMR. Acceptable fit was defined as CFI and TLI ≥ 0.90, RMSEA ≤ 0.08, and SRMR ≤ 0.08. Invariance was supported when nested model comparisons showed non-significant chi-square difference tests (p > 0.05) and changes in fit indices were minimal (ΔCFI ≤ 0.01, ΔRMSEA ≤ 0.015). Average Variance Extracted (AVE) was calculated for each group to assess convergent validity.

Pearson's correlation coefficient was calculated to investigate the intercorrelations between CUDCAS, GAD-7 and AIS. Descriptive statistics (means with standard deviations) were calculated for primary demographic and behavioral variables (age, height, weight, body mass index, and coffee consumption) to summarize the degree of central tendency and variability within our sample.

### 2.5 Recruitment and data collection

Participants were recruited through online advertisements disseminated across multiple social media platforms (e.g., Facebook, Instagram, X, and WhatsApp) with the assistance of trained research volunteers. Recruitment was conducted in three countries: Bahrain, Jordan, and Tunisia, to ensure cross-cultural representation and enhance the generalizability of findings. The study specifically targeted habitual coffee consumers to assess the psychometric properties of the CUDCAS and to explore its associations with insomnia and anxiety symptoms. Inclusion criteria required participants to be adults aged 18 years or above, fluent in Arabic, and in good physical health, with no self-reported history of chronic medical conditions or diagnosed mental disorders.

The sample size (*n* = 523) was determined in accordance with established recommendations for psychometric scale validation. Guidelines typically suggest a ratio of at least 5–10 participants per item, with an absolute minimum of 200 participants for factor analysis (59, 60). With 11 items, our sample exceeded these requirements, yielding over 45 participants per item. Simulation studies further indicate that sample sizes of ≥500 provide excellent power and stability for CFA (61) and are sufficient for robust IRT models (58). Therefore, the present sample was adequate to achieve the study's objectives and ensure reliable estimation of psychometric parameters.

## 3 Results

### 3.1 Participants

Table 1 presents the descriptive statistics for all study variables based on the complete sample of 523 participants, including men (*n* = 101; 19.3%) and women (*n* = 422; 80.7%). They had a mean age of 23.62 years (SD = 7.5), with a positively skewed distribution (skewness = 2.85) and high kurtosis (9.68), indicating a relatively young sample with some older outliers. Referring to their marital status, 435 (83.2%) were single and 88 (16.8%) were married. Anthropometric measurements showed participants had an average height of 161.43 cm (SD = 8.65) and weight of 61.07 kg (SD = 15.23), resulting in a mean body mass index of 23.29 kg/m^2^ (SD = 4.78), which falls within the normal weight range according to WHO classifications.

**Table 1:** Descriptive statistics of the study variables (*N* = 523).

**Variable**	**Mean**	**SD**	**Median (IQR)**	**Skewness**	**Kurtosis**
Age (years_)	23.62	7.5	21 (5)	2.85	9.68
Ht (cm)	161.43	8.65	160 (11.5)	0.63	0.29
Wt (kg)	61.07	15.23	58 (20)	1.03	1.81
BMI (kg/m^2^)	23.29	4.78	2 (0)	0.72	0.52
Q1	1.97	0.6	2 (0)	0.01	−0.25
Q2	2.04	0.57	2 (0)	0	0.05
Q3	1.92	0.6	2 (1)	0.03	−0.22
Q4	1.83	0.61	2 (1)	0.11	−0.46
Q5	1.83	0.59	2 (0)	0.05	−0.29
Q6	1.9	0.6	2 (0)	0.04	−0.27
Q7	1.98	0.64	2 (1)	0.02	−0.54
Q8	1.64	0.62	2 (0)	0.43	−0.66
Q9	1.95	0.61	2 (1)	0.03	−0.29
Q10	1.71	0.57	2 (1)	0.09	−0.55
Q11	1.72	0.59	5 (2)	0.17	−0.56
CUDCAS	4.75	2.11	257 (160)	0.43	1.02
Caffeine intake (mg/day)	258.04	118.34	6 (5)	0.18	−0.27
AIS	6.15	3.03	9 (9)	0.2	−0.41
GAD	9.18	4.77	21 (6)	0.12	−0.67

Ht, Height; Wt, Weight; BMI, Body Mass Index; Q1-Q11, Questionnaire items; CUDCAS, Coffee Use Disorder and Coffee Addiction Scale; Caffeine intake, Caffeine intake from Coffee per day in milligram; AIS, Athens Insomnia Scale; GAD-7, Generalized Anxiety Disorder Scale.

### 3.2 Descriptive statistics

Regarding the questionnaire items (Q1–Q11), mean scores ranged from 1.64 (Q8) to 2.04 (Q2), with standard deviations between 0.57 and 0.64, indicating relatively low variability in responses. Most items showed minimal skewness (ranging from −0.03 to 0.43) and negative kurtosis values (ranging from −0.66 to 0.05), suggesting approximately normal distributions with slightly flattened peaks compared to a normal distribution.

The CUDCAS showed a mean score of 4.75 (SD = 2.11) with positive skewness (0.43) and kurtosis (1.02), indicating a slight right-skewed distribution. Daily caffeine intake from coffee averaged 258.04 mg (SD = 118.34) with minimal skewness (0.18) and slight negative kurtosis (-0.27), suggesting a relatively normal distribution. Sleep quality, as measured by the AIS, had a mean score of 6.15 (SD = 3.03), while anxiety levels measured by the GAD-7 scale averaged 9.18 (SD = 4.77). Both AIS and GAD-7 scores showed low positive skewness (0.2 and 0.12, respectively) and negative kurtosis (−0.41 and −0.67, respectively), indicating approximately normal distributions with slightly flattened peaks (Table 1).

### 3.3 Internal consistency analysis

Table 2 presents the internal consistency analysis of the CUDCAS. The scale demonstrated excellent internal consistency with an overall Cronbach's α of 0.86 and McDonald's ω of 0.86, both exceeding the recommended threshold of 0.70 for acceptable reliability. Item-rest correlations ranged from 0.31 (Q10) to 0.65 (Q9), with most items showing moderate to strong correlations with the total scale score. Notably, Q10 exhibited the lowest item-rest correlation (0.31), which is considered adequate but approaching the lower threshold for acceptable item performance.

**Table 2:** Internal consistency of the Coffee Use Disorder and Coffee Addiction Scale (CUDCAS) (*N* = 523).

**Variable**	**Item-rest correlation**	**Cronbach's α**	**McDonald's ω**
Q1	0.45	0.85	0.85
Q2	0.61	0.84	0.84
Q3	0.60	0.84	0.84
Q4	0.59	0.84	0.84
Q5	0.48	0.85	0.85
Q6	0.59	0.84	0.84
Q7	0.56	0.84	0.84
Q8	0.56	0.84	0.84
Q9	0.65	0.83	0.84
Q10	0.31	0.86	0.86
Q11	0.57	0.84	0.84
CUDCAS	Not applicable	0.86	0.86

CUDCAS, Coffee Use Disorder and Coffee Addiction Scale; Cronbach's α, A measure of internal consistency reliability; McDonald's ω, An alternative measure of reliability that can be more informative in certain contexts; Item-rest correlation, The correlation between an individual item and the total score of the remaining items.

The “alpha if item deleted” analysis revealed that removing most items would result in minimal changes to the overall reliability, with Cronbach's α values ranging from 0.83 to 0.86 across individual items. The highest alpha values (0.86) were observed when Q1, Q5, or Q10 were hypothetically removed, suggesting these items contribute less to the overall internal consistency compared to other items. Conversely, removing Q9 would result in the lowest alpha value (0.83), indicating this item contributes most strongly to the scale's internal consistency. McDonald's ω values closely paralleled the Cronbach's α results, ranging from 0.84 to 0.86, providing additional confirmation of the scale's reliability. These findings support the use of the CUDCAS as a psychometrically sound instrument for measuring coffee use disorder and addiction symptoms in the study population.

### 3.4 Exploratory Factor Analysis (EFA)

Conducting EFA was not strictly necessary in the current study since the CUDCAS was developed based on the well-established symptom criteria outlined in the DSM-5 for substance use disorders. However, we performed an EFA to empirically examine the underlying structure and assess item performance within the target population. EFA was carried out on a subsample of 100 participants using the maximum likelihood extraction method with promax rotation. Sampling adequacy was supported by a KMO measure of 0.800 for the overall scale, with item-level KMO values ranging from 0.598 to 0.893, all exceeding the acceptable minimum of 0.50. The Bartlett's Test of Sphericity yielded a highly significant result (χ^2^ = 298.752, df = 55, *p* < 0.001), confirming the factorability of the correlation matrix. Factor loadings ranged from 0.358 to 0.751, indicating moderate to strong associations between items and the extracted factor. Fit indices further supported the model, with CFI and TLI values approaching 0.90, and RMSEA and SRMR values falling near or below the conventional cutoff of 0.08, suggesting an acceptable model fit.

### 3.5 Confirmatory Factor Analysis (CFA)

Table 3 presents the results of the CFA conducted to examine the factorial structure of the CUDCAS using maximum likelihood extraction. The analysis revealed that all 11 items loaded significantly onto a single factor (all *p* < 0.001), supporting the unidimensional structure of the scale. Factor loadings ranged from 0.18 (Q10) to 0.44 (Q9), with standardized estimates ranging from 0.32 (Q10) to 0.72 (Q9). The majority of items demonstrated moderate to strong factor loadings, with Q9 showing the highest loading (standardized estimate = 0.72), followed by Q2 (0.68) and Q3 (0.67). Q10 exhibited the weakest factor loading (standardized estimate = 0.32), consistent with its lower item-rest correlation observed in the reliability analysis.

**Table 3:** Factorial structure of the Coffee Use Disorder and Coffee Addiction Scale (CUDCAS) (*N* = 523).

**Factor**	**Indicator**	**Estimate**	**SE**	**Z**	***p***	**Stand. estimate**
CUDCAS	Q1	0.30	0.03	11.2	< 0.001	0.49
	Q2	0.39	0.02	16.5	< 0.001	0.68
	Q3	0.40	0.02	16.37	< 0.001	0.67
	Q4	0.39	0.03	15.17	< 0.001	0.63
	Q5	0.30	0.03	11.57	< 0.001	0.51
	Q6	0.39	0.02	15.83	< 0.001	0.65
	Q7	0.38	0.03	13.95	< 0.001	0.59
	Q8	0.38	0.03	14.59	< 0.001	0.61
	Q9	0.44	0.02	17.89	< 0.001	0.72
	Q10	0.18	0.03	7.03	< 0.001	0.32
	Q11	0.36	0.02	14.57	< 0.001	0.61

Confirmatory factor analysis using Maximum Likelihood Extraction. RMSEA: 0.07; CFI: 0.92; TLI: 0.90; SRMR: 0.04; AIC: 8,920.19; BIC: 9,060.76. RMSEA, Root Mean Square Error of Approximation; CFI, Comparative Fit Index; TLI, Tucker-Lewis Index; SRMR, Standardized Root Mean Square Residual.

Values indicate a good model fit, with RMSEA below 0.06 and CFI/TLI above 0.90.

The overall model fit indices indicated good fit to the data. The RMSEA was 0.07, which is below the recommended threshold of 0.08 for good fit. The CFI was 0.92 and the TLI was 0.90, both meeting or exceeding the recommended cutoff of 0.90 for acceptable fit. The SRMR was 0.04, below the ideal threshold of 0.08. The Akaike Information Criterion AIC of 8,920.19 and BIC of 9,060.76 provide reference values for model comparison purposes. These results provide strong evidence for the unidimensional factorial structure of the CUDCAS and support its construct validity in the current sample.

### 3.6 Item Response Theory (IRT) analysis

Table 4 presents the IRT analysis of the CUDCAS using the Rating Scale Model with Delta-Tau parameterization of the Partial Credit Model, estimated via Marginal Maximum Likelihood Estimation (MMLE). The item difficulty measures ranged from −4.00 (Q2) to −2.29 (Q8), indicating that Q2 was the most difficult item to endorse (requiring higher levels of coffee use disorder symptoms) while Q8 was the easiest item to endorse. All items showed consistent standard errors of measurement (S.E. = 0.09), indicating similar precision across items.

**Table 4:** Item response theory of the Coffee Use Disorder and Coffee Addiction Scale (CUDCAS) (*N* = 523).

**Item statistics of the rating scale model**			**Delta-tau parameterization of the partial credit model**		
**Item**	**Measure**	**S.E. measure**	τ**1**	τ**2**	τ**3**
Q1	−3.68	0.09	−28.23	12.17	16.06
Q2	−4.00	0.09	−27.32	11.53	15.79
Q3	−3.47	0.09	−29.02	12.51	16.51
Q4	−3.11	0.09	−27.48	11.85	15.62
Q5	−3.09	0.09	−28.92	12.39	16.53
Q6	−3.40	0.09	−28.06	12.06	16.00
Q7	−3.73	0.09	−27.78	12.16	15.62
Q8	−2.29	0.09	−27.8	12.2	15.60
Q9	−3.59	0.09	−28.03	12.09	15.94
Q10	−2.59	0.09	−30.48	13.02	17.46
Q11	−2.63	0.09	−29.49	12.72	16.76

Results are estimated using Marginal Maximum Likelihood Estimation (MMLE); The eRm R package was utilized for the person-item mapping.

The threshold parameters (τ1, τ2, τ3) represent the difficulty of moving from one response category to the next for each item. The first threshold (τ1) values ranged from−30.48 (Q10) to−27.32 (Q2), indicating the difficulty of moving from the lowest to the second response category. The second threshold (τ2) values ranged from 11.53 (Q2) to 13.02 (Q10), representing the difficulty of moving from the second to the third response category. The third threshold (τ3) values ranged from 15.60 (Q8) to 17.46 (Q10), indicating the difficulty of moving from the third to the highest response category.

The IRT analysis revealed that Q8 had the lowest difficulty measure (−2.29), suggesting it captures lower levels of coffee use disorder symptoms and is more easily endorsed by participants. Conversely, Q2 had the highest difficulty measure (−4.00), indicating it requires higher symptom levels for endorsement. Q10 showed distinctive threshold patterns with the most extreme τ1 (−30.48) and τ3 (17.46) values, suggesting unique response patterns compared to other items. These findings provide detailed psychometric information about individual item functioning and support the use of IRT-based scoring for more precise measurement of coffee use disorder symptoms.

### 3.7 Multi-group confirmatory factor analysis testing gender invariance

Table 5 presents the multi-group confirmatory factor analysis examining gender invariance of the CUDCAS scale, which demonstrated strong support for measurement invariance across male and female groups. The configural invariance model, which tested whether the same factor structure held across groups, showed acceptable fit (χ^2^ = 255.74, df = 88, CFI = 0.90, TLI = 0.88, RMSEA = 0.07, SRMR = 0.05). The metric invariance model, constraining factor loadings to be equal across groups, showed improved fit indices (CFI = 0.91, TLI = 0.90, RMSEA = 0.08) with a non-significant chi-square difference test (Δχ^2^ = 1.81, Δdf = 10, *p* > 0.05), indicating that factor loadings were equivalent across gender groups. The scalar invariance model, additionally constraining item intercepts to equality, maintained excellent fit (CFI = 0.91, TLI = 0.91, RMSEA = 0.08) with a non-significant chi-square difference test compared to the metric model (Δχ^2^ = 10.29, Δdf = 10, *p* > 0.05). These findings support full scalar invariance, indicating that the CUDCAS scale measures the same construct equivalently across male and female participants, and that meaningful comparisons of factor means between gender groups are justified. The Average Variance Extracted (AVE) values were similar across groups (Female = 0.35, Male = 0.40), further supporting the psychometric equivalence of the scale across gender.

**Table 5:** Model fit indices for multi-group confirmatory factor analysis testing gender invariance.

**Model**	**χ^2^**	**df**	**CFI**	**TLI**	**RMSEA**	**SRMR**	**AIC**	**BIC**	**Model comparison**	***Δχ* ^2^**	**Δdf**	***p*-value**
Configural	255.74	88	0.90	0.88	0.07	0.05	8,956.51	9,237.64	-	-	-	-
Metric	257.55	98	0.91	0.90	0.07	0.05	8,938.33	9,176.87	Metric vs. Configural	1.81	10	> 0.05
Scalar	267.84	108	0.91	0.91	0.08	0.05	8,928.61	9,124.55	Scalar vs. Metric	10.29	10	> 0.05

CFI, Comparative Fit Index; TLI, Tucker-Lewis Index; RMSEA, Root Mean Square Error of Approximation; SRMR, Standardized Root Mean Square Residual; AIC, Akaike Information Criterion; BIC, Bayesian Information Criterion.

### 3.8 Correlations

Table 6 presents the correlations among the primary study variables, including the CUDCAS, daily caffeine intake, insomnia symptoms (AIS), and anxiety symptoms (GAD-7). All correlations were statistically significant at *p* < 0.001, indicating robust associations between the variables. The strongest correlation was observed between caffeine intake and insomnia symptoms (r = 0.70), suggesting a strong positive relationship between daily caffeine consumption and sleep disturbances. This was followed by the correlation between insomnia and anxiety symptoms (r = 0.69), indicating a strong association between sleep problems and anxiety levels.

**Table 6:** Correlation between Coffee Use Disorder and Coffee Addiction Scale (CUDCAS), daily caffeine intake in milligrams, insomnia symptoms, and anxiety symptoms (*N* = 523).

**Variable**	**CUDCAS**	**Caffeine**	**AIS**	**GAD-7**
CUDCAS	—			
Caffeine	0.54^*^	—		
AIS	0.37^*^	0.70^*^	—	
GAD-7	0.32^*^	0.50^*^	0.69^*^	—

CUDCAS, Coffee Use Disorder and Coffee Addiction Scale; Caffeine intake, Caffeine intake from Coffee per day in milligram; AIS, Athens Insomnia Scale; GAD-7, Generalized Anxiety Disorder Scale. ^*^p < 0.001.

The CUDCAS demonstrated moderate to strong correlations with all other variables. The strongest association was with daily caffeine intake (r = 0.54), providing evidence for the convergent validity of the scale, as higher coffee use disorder symptoms were associated with greater caffeine consumption. The CUDCAS also showed moderate correlations with insomnia symptoms (r = 0.37) and anxiety symptoms (r = 0.32), suggesting that coffee use disorder symptoms are associated with both sleep disturbances and anxiety, though these relationships were weaker than those involving caffeine intake directly.

Caffeine intake showed strong correlations with both insomnia (r = 0.70) and anxiety symptoms (r = 0.50), indicating that higher daily caffeine consumption is associated with greater sleep problems and anxiety levels. The correlation pattern suggests that caffeine intake may serve as a mediating factor in the relationships between coffee use disorder symptoms and various psychological and physiological outcomes. These findings support the theoretical framework underlying the study and provide evidence for the interconnected nature of coffee use disorder symptoms, caffeine consumption, sleep disturbances, and anxiety.

## 4 Discussion

The present study developed and evaluated the psychometric properties of CUDCAS, based on the DSM-5 criteria of substance use disorder (23). The results provide strong support that the CUDCAS is a consistent, valid, and psychometrically sound measure of problematic coffee use and behavioral addiction symptoms.

The item-level descriptive statistics showed acceptable variability and generally normal distributions for all items (mean scores between 1.64 and 2.04, skewness and kurtosis values well within acceptable limits). These descriptive statistics suggest the items were well-constructed and have a reasonable range of symptoms to capture symptom intensity. The total CUDCAS score had a slight positive skew, which is consistent with our expectations that only a smaller number of participants would report higher levels of coffee use disorder symptoms, similar to past research describing right skewed distributions with behavioral measures of addiction (28, 38).

Internal consistency analysis showed excellent reliability for the total scale, with Cronbach's alpha and McDonald's omega values of 0.86, well above the acceptable level of 0.70 (62). The item-test correlations ranged from 0.31 to 0.65, with the range from 0.31 to 0.47 indicating a moderate association to 0.54–0.65 indicating a strong association between the items and the overall composite construct. It is worth noting that this instrument had the lowest item-rest correlation and the weakest CFI loading for Item Q10 (“Tolerance”). This is consistent with previous studies of substance use assessments, where physiological symptoms, including tolerance or withdrawal, are quoted less frequently in behavioral paradigms of addiction (63).

CFA provided strong evidence of a unidimensional structure for the CUDCAS, with all items loading significantly on a single latent factor (*p* < 0.001). Fit indices met or were very close to conventional guidelines for acceptable to good fit and provided confirmation of the structure intended by the scale. These findings are consistent with the factorial patterns in the validation of the Caffeine Use Disorder Questionnaire (15), thereby reinforcing the CUDCAS as a brief and meaningful measurement tool.

We can notice from our IRT analysis using the Partial Credit Model that our psychometric evaluation was enhanced by obtaining item-specific estimates of precision. Item difficulty parameters ranged from −4.00 (Q2) to −2.29 (Q8), providing evidence of item good item spread across the latent trait. Q2, which referenced very severe forms of coffee addiction, was demonstrated to be the hardest to endorse, and Q8, which referenced symptoms that were less stigmatized or commonly accepted, was demonstrated to be the easiest to endorse. The pattern of these item difficulties corresponded with the symptom endorsement hierarchies from prior behavioral disorder scales (64) and the relatively consistent measurement error seen across items (SE = 0.09) provides strong evidence of the CUDCAS's precision across levels of the latent trait continuum.

The establishment of scalar invariance for the CUDCAS scale across gender groups represents a significant psychometric contribution, providing empirical evidence that the instrument functions equivalently for both male and female participants. This finding is particularly important given the potential for gender differences in career decision-making processes and academic self-perceptions. The achievement of full measurement invariance indicates that observed differences in CUDCAS scores between males and females can be attributed to true differences in the underlying construct rather than measurement bias or differential item functioning. The relatively low but acceptable AVE values (0.35–0.40) suggest that while the scale demonstrates adequate convergent validity, future research might benefit from refining items to enhance internal consistency. These results support the use of CUDCAS in comparative research across gender groups and strengthen confidence in gender-based analyses of career decision-making difficulties. The findings also contribute to the broader literature on measurement invariance in psychological assessment, demonstrating the feasibility of achieving scalar invariance even with complex constructs related to career development.

The intercorrelational analysis demonstrated parallel associations to the CUDCAS's construct validity. The CUDCAS was moderately to strongly correlated with daily caffeine intake, insomnia, and anxiety all statistically significant. These findings are echoed in previous literature reporting a relationship between problematic caffeine intake and sleep disruptions (65) as well as anxiety symptoms (38, 63). The strongest association in the correlation matrix was between caffeine intake and insomnia (r = 0.70). This connection highlights the clinical validity of assessing problematic caffeine consumption using a disorder-based approach.

The addition of milk to coffee is considered an essential factor as it could affect the therapeutic potential and bioavailability of coffee's beneficial components, and this factor should be borne in mind as the CUDCAS is applied in the future. It is known that milk proteins, especially casein, interact with and chelate polyphenolic compounds (e.g., chlorogenic acids, flavonoids) (66–68), reducing their antioxidant potential and bioavailability by up to 50% (69). Furthermore, these protein-polyphenol bindings can negatively influence the bioavailability of the essential minerals naturally present in coffee, that is, iron, magnesium, potassium, and manganese, by forming insoluble complexes (70, 71). Dietary calcium in milk can also interfere with iron absorption from coffee, which is of particular concern among coffee drinkers who make coffee their primary beverage of choice or who have an iron deficiency (72, 73). Lastly, the pharmacokinetics of caffeine in combination with milk fat may also regulate the intensity of caffeine's psychoactive effects (74), thereby contributing to changes in the onset and duration of its psychoactive properties. These suggest that coffee mixtures containing milk may result in either physiological or psychological differences outcomes compared to black coffee, which may determine CUDCAS scores and the symptom prevalence in coffee use disorder (74, 75). It is also crucial to have a comprehensive understanding of these variables related to preparation to improve the clinical application of the CUDCAS, and to develop more accurate measures for addressing the harmful coffee drinking habits.

The effects of milk addition to coffee on hormonal attributes and metabolic reaction are not highlighted properly in the literature. Casein and whey proteins in milk have been demonstrated to increase secretion of insulin-like growth factor-1 (IGF-1) and the regulation of insulin sensitivity, thus changing the impact of coffee on metabolism (76). Caffeine and milk proteins could also interactively alter cortisol reactivity unlike that of black coffee, which could possibly recalculate stress-hormone schematic and the circadian rhythm (35, 77). Moreover, the presence of lactose in milk could alter glucose metabolism and affect the causes of caffeine to affect blood sugar control, a process that is uniquely more important to individuals with diabetes or the metabolic disorder (78–80).

Our results for the meaningful correlations between CUDCAS scores with insomnia and anxiety symptoms are bolstered by the growing meta-analytic and empirical literature. A recent meta-analysis showed that high caffeine-consuming individuals have significantly greater anxiety than lower intake individuals (16). A recent observational research also supports an association between significantly more caffeine exposure and psychological distress, even when insomnia was not explicitly measured (17). This supports our viewpoint on the CUDCAS's convergent validity. Importantly, population-based studies consistently identify insomnia as a common outcome of coffee use; in one report, more than half of adult respondents in Jazan (Saudi Arabia) attributed their inability to sleep to coffee consumption (17). This adds further face validity to the CUDCAS symptom assessment of sleep-related outcomes. Overall, we note that by contextualizing our findings with the burgeoning evidence base in this growing literature, we highlight the relevance of the CUDCAS and its timeliness as a measure of maladaptive coffee-related use.

This study found no statistically significant differences among coffee use disorder or coffee addiction scores based on the age, sex, or marital status of participants. These results may imply that the existence of problematic coffee consumption could be a demographically widespread phenomenon and not limited to age groups or social categories. Some earlier studies (28, 38) found similar results with no significant sex or age differences in caffeine dependence or withdrawal severity. Additionally, Ágoston et al. (15) found no significant demographic predictors of caffeine use disorder severity, corroborating the notion that problematic caffeine use does not discriminate between population segments (15).

Though shorter screening tools are useful for quick assessments, we intended to include the full range of diagnostic criteria in this initial validation to optimize construct validity. Therefore, the 11-item CUDCAS is directly comparable to DSM-5 substance use disorder criteria and the ICD-11 classification of harmful caffeine use. Subsequent studies should explore the viability of a shorter screening version of CUDCAS (e.g., CUDCAS-SF) that is sufficiently robust in terms of psychometric properties as with other substance-use screening tools (e.g., the AUDIT-C). When considered collectively, the findings support the theoretical construct of caffeine/coffee addiction as a maladaptive pattern of behavior that can be reliably measured using DSM-5-consistent criteria. The CUDCAS fills an important gap in measurement space using psychometric instrumentation by providing a coffee specific scale to measure consumption and behavioral dependence.

### 4.1 Limitations

The present research demonstrated reliable psychometric properties of the CUDCAS with a few limitations. The present study did not assess participants' smoking habits or overall dietary consumption, which may interact with their caffeine metabolism and consumption. Smoking, for example, may induce the cytochrome P450 1A2 enzyme, which speeds up caffeine metabolism and ultimately leads to elevations in caffeine consumption rates to attain psychoactive-like effects similar to non-smokers (81, 82). Not controlling for whether someone is a smoker may have affected caffeine intake levels and caffeine-related symptoms, such as sleep or anxiety.

Similarly, dietary behaviors—particularly the intake of other caffeine-containing products (e.g., chocolate, energy drinks, tea), and overall nutritional habits—may have an impact on physiological and psychological responses to coffee consumption (83). These uncontrolled factors may partially explain some of the unexplained variance in outcomes associated with insomnia and anxiety symptoms, and in turn limits the specific nature of the findings from the CUDCAS. Follow-up studies should capture smoking status and nicotine dependence, as well as complete dietary intake, to receive more specific contextualization of coffee use disorder and associated variables. Including these covariates will increase discriminant validity and ecological validity of the scale applicable to different populations.

### 4.2 Future implications for research and clinical practice

The development and validation of the CUDCAS has significant implications. First, clinicians and researchers now have a psychometrically sound measure of the severity of these coffee use disorder symptoms that, in many cases, may have gone unrecognized in clinical screening due in large part to coffee consumption being normalized in societies globally. Future studies may utilize the scale to identify subclinical levels of problematic use in populations that may be more susceptible to dependence, including students, shift workers, and those with anxiety or sleep disorders.

In the present study, self-reported caffeine intake was selected as the primary measure for establishing convergent validity of the CUDCAS, as it effectively captures habitual consumption patterns and aligns with established epidemiological approaches in caffeine research (64, 84). This method demonstrated robust associations with CUDCAS scores, reflecting chronic, problematic use rather than transient exposure. In contrast, biomarkers such as serum or salivary caffeine levels are more indicative of acute toxicity or immediate physiological effects, given caffeine's short half-life of approximately 4 h and substantial interindividual variability in metabolism influenced by factors like genetics, age, and lifestyle (65). While these biomarkers may not reliably represent long-term patterns of coffee use disorder, they hold promise for future multimethod validations, particularly if advanced caffeine monitoring technologies—such as wearable sensors or real-time assays—become available to track and adjust serum levels throughout the day, thereby enhancing the ecological validity and precision of assessments in clinical settings.

Secondly, the CUDCAS's unidimensionality and sound psychometric properties encourage cross-cultural adaptations and longitudinal studies. In future research, the CUDCAS should be validated in various languages and demographic contexts. Thirdly, in clinical practice, the CUDCAS could be used as a screening tool in primary care, sleep clinics, or behavioral health programs. It is brief and tailored to DSM diagnostic criteria allowing for identifying, referring, and intervening when someone may not recognize their coffee use as problematic.

CUDCAS is a clinically promising tool in nutritional assessment and dietary counseling practice that originated from excessive coffee drinking. Registered dietitians and nutritionists can use this scale to identify individuals whose coffee consumption may complicate the achievement of optimal nutritional status and compliance with their diets. High CUDCAS are predictive of problematic coffee use, which has been linked to reduced appetite, irregular meal timing, and an increased risk of developing deficiencies, both through its ability to suppress the appetite and through interference with nutrient availability due to coffee. In addition, high CUDCAS is usually associated with poor dietary quality, with occult increased coffee consumption commonly crowding out high-quality food and beverage selections. These results can inform specific nutritional interventions, enabling health professionals to establish individualized programs that help individuals overcome coffee addiction while ensuring the body receives the necessary amount of essential nutrients needed and enforcing better eating habits. Such utility is particularly important in clinical nutrition services, due to the potential contribution of coffee addiction to underlying nutritional deficiencies or limitations of therapeutic dietary prescription.

Lastly, future research endeavors should also examine the potential utility of the CUDCAS as a predictor of health-related outcomes, such as cardiovascular risk, mood disorders, or even academic/work functioning. The use of biological markers (e.g., cortisol levels, sleep architecture) could also expand the ecological validity of the index. In summary, the CUDCAS is a new and timely addition to the field of substance-related and behavioral addictions literature that allows for a strong foundation for both research and practitioner practice.

## 5 Conclusions

In this study, we presented and evaluated the psychometric properties of a new self-report measure aligned with the DSM-5, the Coffee Use Disorder and Coffee Addiction Scale (CUDCAS), to measure problematic coffee consumption. The data indicate that the CUDCAS offers a reliable, valid, and unidimensional measure that assesses the array of behavioral and physiological symptoms related to coffee use disorder. Findings from the internal consistency, confirmatory factor analysis, and item response theory (IRT) analyses offer convergent evidence of the degree of structural consistency and measurement precision throughout the scale. Further, finding significant correlations with caffeine consumption, insomnia, and anxiety symptoms, offers construct validity evidence for the CUDCAS and further suggests its utility in research and clinical settings.

The CUDCAS fills a significant gap in the available assessment tools through firm psychometric assessment and the operationalization of coffee-specific addiction criteria. Its potential utility is significant, particularly considering coffee's global normalization of consumption and increased recognition of caffeine-related problems in health psychology and psychiatry. The scale provides a strong basis to begin identifying future research, screening, and intervention options. The CUDCAS can potentially facilitate public health initiatives aimed at improving knowledge of and mitigating the behavioral and psychological correlates associated with extreme coffee consumption in a manner like other substance use research.

## Data Availability

The raw data supporting the conclusions of this article will be made available by the authors, without undue reservation.
