# Supplementary material for: The development and validation of Coffee Use Disorder and Coffee Addiction Scale (CUDCAS) and its correlation with insomnia and anxiety symptoms
Source: Front Nutr. 2025 Nov 17;12:1674097. doi: 10.3389/fnut.2025.1674097 (PMC12665531; doi:10.3389/fnut.2025.1674097)
Supplement: Supplementary file 1 [file Data_Sheet_1.docx]

**Supplementary Material:**

**Coffee Use Disorder and Coffee Addiction Scale (CUDCAS)**

**Instructions:** For each statement, indicate how often it applies to your coffee consumption over the past three months. The questions exclude periods of social (e.g. festive holiday), occupational (e.g. exams or competitions), or religious (e.g. Ramadan fasting) practices. The statements focus on drinking coffee, but also extends to using lozenges and dissolvable coffee pouches.

| **Item *(DSM-5 symptom)*** | **Never** | **Sometime** | **Always** |
| --- | --- | --- | --- |
| 1. I drink more coffee or consume it in stronger versions. *(Larger Amounts)* |  |  |  |
| 1. I have tried to cut down or stop drinking coffee, but I can't. *(Unsuccessful Attempts to Quit)* |  |  |  |
| 1. I spend a lot of time getting coffee, drinking it, or recovering from its effects/side effects. *(Time Spent)* |  |  |  |
| 1. I feel strong cravings and urges to drink coffee. *(Cravings)* |  |  |  |
| 1. My coffee drinking interferes with my responsibilities at work, home, or school e.g. late arrival to work due to stopping at café to drink or pick coffee. *(Neglecting Responsibilities)* |  |  |  |
| 1. I continue to drink coffee even when it causes problems in my relationships e.g. arguments with others who express concern about your excessive coffee consumption. *(Relationship Issues)* |  |  |  |
| 1. I have given up important activities because of my coffee consumption e.g. adequate sleep or healthy diet. *(Giving Up Activities)* |  |  |  |
| 1. I drink coffee even when it puts me in risky situations e.g., drinking too much caffeine. *(Dangerous Use)* |  |  |  |
| 1. I continue drinking coffee despite knowing it worsens physical or psychological issues. *(Continued Use Despite Problems)* |  |  |  |
| 1. I need to drink more coffee to feel its effects. *(Tolerance)* |  |  |  |
| 1. I experience withdrawal physical or psychological symptoms that are relieved by drinking coffee. *(Withdrawal)* |  |  |  |

**Scoring**

- **Never = 0**.
- **Sometimes = 0.5.**
- **Always = 1**.

**Suggested Interpretation**

- **Mild Coffee Use Disorder:** <3 symptoms.
- **Moderate Coffee Use Disorder:** 4-6 symptoms.
- **Severe Coffee Use Disorder:** ≥7 or more symptoms.
